# Delivery of Doxorubicin for Human Cervical Carcinoma Targeting Therapy by Folic Acid-Modified Selenium Nanoparticles

**DOI:** 10.3390/ijms19113582

**Published:** 2018-11-13

**Authors:** Yu Xia, Tiantian Xu, Mingqi Zhao, Liang Hua, Yi Chen, Changbing Wang, Ying Tang, Bing Zhu

**Affiliations:** Central Laboratory, Guangzhou Institute of Pediatrics, Guangzhou Women and Children’s Medical Center, Guangzhou Medical University, Guangzhou 510120, China; yuxiadr@126.com (Y.X.); tiantxu@163.com (T.X.); gzwcmczhaomq@126.com (M.Z.); liangzai_hua@126.com (L.H.); chenyizhuxi@sina.com (Y.C.); gzwangcb@sina.com (C.W.); gzwcmctangy@126.com (Y.T.)

**Keywords:** tumor targeting, human cervical carcinoma, folic acid, doxorubicin, nanoparticles

## Abstract

Cancer-specific drug delivery represents an attractive approach to preventing undesirable side effects and increasing the accumulation of the drug in tumors. The surface modification of selenium nanoparticles (SeNPs) with targeting moieties thus represents an effective strategy for cancer therapy. In this study, SeNPs were modified with folic acid (FA), whose receptors were overexpressed on the surface of cancer cells, including human cervical carcinoma HeLa cells, to fabricate tumor-targeting delivery carrier FA-SeNPs nanoparticles. Then, the anticancer drug doxorubicin (DOX) was loaded onto the surface of the FA-SeNPs for improving the antitumor efficacy of DOX in human cervical carcinoma therapy. The chemical structure characterization of FA-Se@DOX showed that DOX was successfully loaded to the surface of FA-SeNPs to prepare FA-Se@DOX nanoparticles. FA-Se@DOX exhibited significant cellular uptake in human cervical carcinoma HeLa cells (folate receptor overexpressing cells) in comparison with lung cancer A549 cells (folate receptor deficiency cells), and entered HeLa cells mainly by the clathrin-mediated endocytosis pathway. Compared to free DOX or Se@DOX at the equivalent dose of DOX, FA-Se@DOX showed obvious activity to inhibit HeLa cells’ proliferation and induce the apoptosis of HeLa cells. More importantly, FA-Se@DOX could specifically accumulate in the tumor site, which contributed to the significant antitumor efficacy of FA-Se@DOX in vivo. Taken together, FA-Se@DOX may be one novel promising drug candidate for human cervical carcinoma therapy.

## 1. Introduction

Human cervical cancer mainly resulted from human papillomavirus (HPV) is one of the most popular cancers in women’s health worldwide, and it is the fifth leading cause of cancer deaths among females [1]. Chemotherapy, photothermal therapy, and radiotherapy are the most common methods for cancer treatments [2]. Chemotherapy is a predominant strategy for cancer treatments due to its high efficacy in comparison with the other treatments [3]. Doxorubicin (DOX) is one very common and effective chemotherapeutic drug for cancer therapy [4]. Nevertheless, clinical application of DOX is limited by its poor water solubility and off-target side effects [5]. Recently, nanomedicine has attracted increasing attention in cancer treatments to overcome the pharmaceutical challenges of hydrophobic anticancer drugs, such as nonspecific biodistribution, off-target toxicity, and poor water solubility [6,7]. Thus, a number of strategies using nanoparticles have been developed for cancer therapy, and most of these therapies are based on the enhanced permeation and retention (EPR) effect [8]. However, uncontrolled drug release and drug delivery to unintended sites may compromise the therapeutic efficacy as well as toxic and side effects [9]. These side effects can be minimized by controlled targeted drug delivery by nanocarriers [10]. Recently, folic acids (FA) have been widely used as active targeting ligands in nanoscale drug delivery systems due to their high specific binding with folate receptors that are overexpressed in various cancer cells, including human cervical carcinoma HeLa cells [11,12].

Selenium nanoparticles (SeNPs) as drug carriers have received a large amount of attention [13,14,15]. For one thing, selenium (Se) as a trace element is very important to human biological processes and involves many physiological functions [16]. For another, Se plays a key role in cancer prevention and immune response [17]. Moreover, SeNPs showed some other advantages, for example the controlled size, potent drug-loading capacity, improved antitumor effect, and low cytotoxicity [18]. SeNPs are regarded as superior to other selenium species owing to their low toxicity, high biological activity, and optical and electronic properties, which have found their way into applications in cancer therapy and diagnosis [19]. Thus, SeNPs gradually developed into one excellent anticancer drug carrier [20]. However, some deficiency, especially the lack of active tumor-targeted capacity, still existed in such a delivery carrier [21]. To obtain a high targeting ability, a lot of tumor-targeted molecules, such as folate, Arg-Gly-Asp (RGD) peptides, and hyaluronic acid, were used for decorating the nanoparticles [22,23,24,25]. Muangsin [23] reported that SeNPs modified with folic acid-N-trimethyl chitosan (TMC-FA) as nanocarriers were used for the delivery of doxorubicin (DOX) to overcome drug-resistant cancer cells, which could obviously enhance the activity of DOX compared to free DOX.

**Scheme 1:**
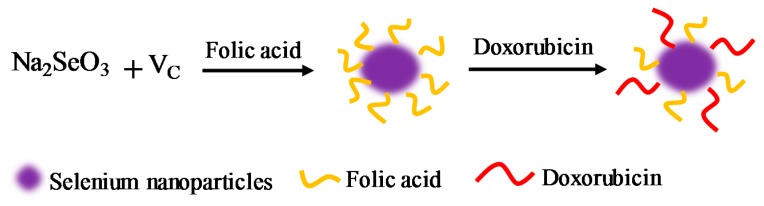
Schematic illustration of the formation of folic acid selenium (FA-Se)@doxorubicin (DOX) nanoparticles.

In this paper, FA was installed onto the surface of SeNPs to prepare tumor-targeted carrier FA-SeNPs. Then, DOX was loaded onto FA-SeNPs nanoparticles to prepare functionalized antitumor nanoparticles FA-Se@DOX (Scheme 1). FA-Se@DOX exhibited significant cellular uptake and the controlled release of DOX in HeLa cells, and could suppress the proliferation, migration, and invasion of HeLa cells in vitro. FA-Se@DOX could also induce the apoptosis of HeLa cells. It is worth noting that FA-Se@DOX obtained more favorable antitumor efficacy in vivo in comparison with Se@DOX or DOX, indicating that FA-Se@DOX holds great potential application in human cervical carcinoma therapy.

## 2. Results and Discussion

### 2.1. Preparation and Characterizations of FA-Se@DOX

**Figure 1:**
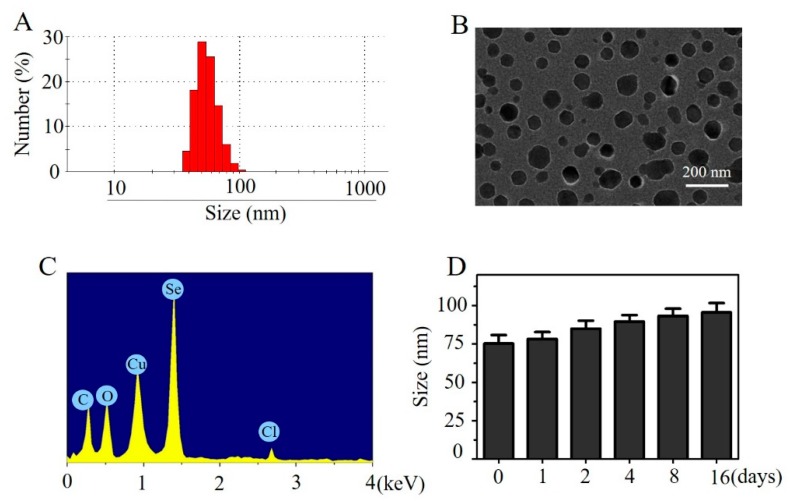
Characterization of FA-Se@DOX nanoparticles. (**A**) Particle size distribution of FA-Se@DOX nanoparticles. (**B**) Representative transmission electron microscopy (TEM) image of FA-Se@DOX nanoparticles. (**C**) Energy-dispersive X-ray (EDX) analysis of FA-Se@DOX nanoparticles. (**D**) Stability observation of FA-Se@DOX nanoparticles in aqueous solution.

One tumor-targeting delivery system, FA-Se@DOX, was fabricated in this paper. The tumor-targeting molecular folic acid (FA) was linked with selenium nanoparticles (SeNPs) to fabricate tumor-targeting delivery carrier FA-SeNPs; then, the antitumor drug doxorubicin (DOX) was loaded to the surface of the FA-SeNPs to prepare the tumor-targeting delivery system FA-Se@DOX. As shown in Figure 1A, the average size of the FA-Se@DOX was 83 nm. The morphology of nanoparticles in the TEM image presented spherical particles with size ranges of 40 nm to 110 nm (Figure 1B). Nanoparticles in the range of 10–100 nm are considered ideal for biomedical use, as they are small enough to avoid uptake by the reticuloendothelial system, but large enough to escape renal filtration. Thus, SeNPs with such sizes are very beneficial for use as drug carriers [19]. The obvious signal of a Se atom and a typical Cl atom signal of DOX⋅HCl appeared in elemental compositions of FA-Se@DOX (Figure 1C), indicating that DOX were successfully loaded to the surfaces of SeNPs. The size distribution of FA-Se@DOX in Figure 1D showed that FA-Se@DOX were stable with small sizes (<100 nm) for 16 days. The above data indicated that FA-Se@DOX were successfully synthesized and exhibited good stability in a water solution.

**Figure 2:**
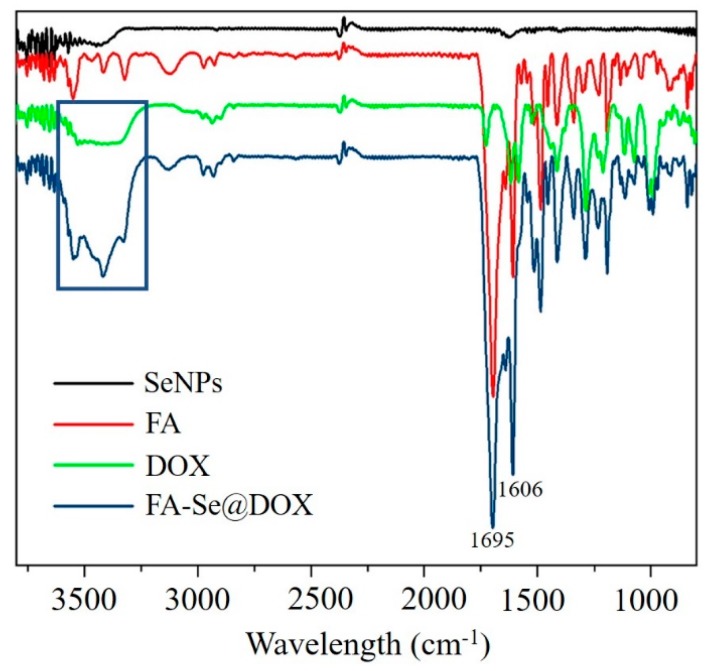
Fourier transform infrared spectroscopy (FTIR) spectra of SeNPs, FA, DOX, and FA-Se@DOX.

The Fourier transform infrared (FTIR) spectrums of FA-Se@DOX, DOX, SeNPs, and FA are shown in Figure 2; typical peak of SeNPs also appeared in the spectrum of FA-Se@DOX. The peaks at 1695 cm^−1^ and 1606 cm^−1^ were corresponded to the carboxyl band of FA. After loading FA to SeNPs, typical carboxyl bands (1697 cm^−1^ and 1607 cm^−1^) also appeared in spectrum of FA-Se@DOX, indicating successful linking between SeNPs and FA via carboxyl bonds. The peak at ~3370 cm^−1^ (blue pane) that was assigned to the characteristic hydroxyl group of DOX existed in the spectrum of FA-Se@DOX, verifying effective linking between DOX and FA-SeNPs.

### 2.2. Cellular Uptake Studies

**Figure 3:**
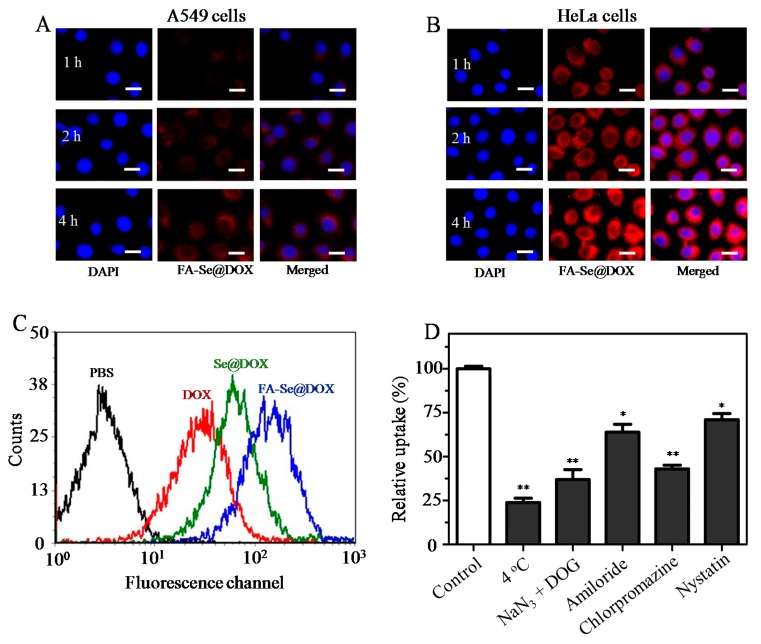
(**A**) Cellular uptake of FA-Se@DOX (red fluorescence) was visualized by red fluorescence from DOX in A549 cells (**A**) and HeLa cells (**B**). Nucleus was indicated by blue fluorescence. Scale bar is 40 μm. (**C**) The cellular uptake efficiency of DOX, Se@DOX, and FA-Se@DOX was analyzed by flow cytometry. (**D**) Effects of endocytosis inhibitors and temperature on the internalization of FA-Se@DOX. * *p <* 0.05, ** *p* < 0.01 vs. control group.

The drug delivery efficiency is closely related to cellular uptake [26]. A high cellular uptake of drugs can result in effective treatment efficacy. It has been reported that folate receptors (FAR) are overexpressed in various cancers, including cervical cancer, breast cancer, brain cancer, and colon cancer [27]. However, A549 cells were usually used as folate receptor-negative control cells [28]. Thus, we aimed to detect the effects of FAR-mediated cellular uptake in HeLa cells and A549 cells. Selective FAR-mediated cellular uptake of FA-Se@DOX between HeLa cells and A549 cells was confirmed by fluorescence microscope. As shown in Figure 3A,B, the cellular uptake of FA-Se@DOX in HeLa cells was stronger than that in A549 cells at the same condition, verifying a FAR-mediated specific uptake between HeLa cells and A549 cells. Furthermore, the cellular uptake of free DOX, passive tumor-targeting nanoparticles Se@DOX, and active tumor-targeting nanoparticles FA-Se@DOX were quantificationally analyzed in HeLa cells using flow cytometry. As shown in Figure 3C, both Se@DOX and FA-Se@DOX exhibited higher cellular uptake than free DOX, indicating that loading DOX onto selenium nanoparticles improved the cellular uptake of DOX in HeLa cells. The uptake of DOX from FA-Se@DOX is relatively higher than that from Se@DOX, which can be explained by FAR-mediated targeting contributing to the improved cellular uptake of FA-Se@DOX in HeLa cells.

It has been reported that nanoparticles can enter the cells in an energy-dependent endocytotic way [29]. The incubation of HeLa cells at 4 °C or pretreated with NaN_3_/DOG markedly reduced the cellular uptake of nanoparticles (Figure 3D), indicating that the endocytosis of FA-Se@DOX nanoparticles is an active energy-dependent process. The cells’ endocytosis mainly includes three pathways, including clathrin-mediated endocytosis, caveolae-mediated endocytosis, and macropinocytosis. To examine the endocytosis mechanism of FA-Se@DOX in HeLa cells, different endocytosis inhibitors were used to study the effects of FA-Se@DOX on cellular uptake. Amiloride, nystatin, and chlorpromazine are usually used to inhibit micropinocytosis, caveolae-mediated endocytosis, and clathrin-associated endocytosis, respectively. After pretreating with amiloride or nystatin, the cellular uptake of FA-Se@DOX was decreased by 36.1% and 29%, respectively. Nevertheless, pretreatments with chlorpromazine resulted in about a 57.2% decrease in the cellular uptake of FA-Se@DOX, suggesting that clathrin-associated endocytosis mainly contributed to the internalization of FA-Se@DOX.

### 2.3. In Vitro Release of DOX

**Figure 4:**
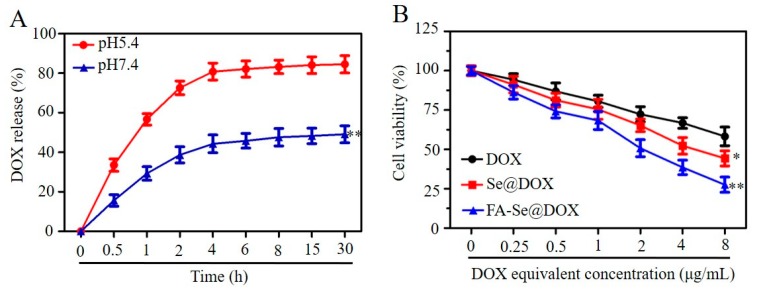
(**A**) In vitro release of DOX from FA-Se@DOX nanoparticles. ** *p <* 0.01 vs. pH 5.4 group. (**B**) In vitro cytotoxicity of DOX, Se@DOX, and FA-Se@DOX on HeLa cells. * *p <* 0.05, ** *p* < 0.01 vs. DOX group.

The two types of pH values (pH 7.4 and 5.4) were used to simulate the normal physiological environment and the cancer cell microenvironment, respectively [30]. The release profiles of DOX from FA-Se@DOX nanoparticles were shown in Figure 4A; there was a noteworthy burst drug release during the initial 4 h in both pH values. It was worth noting that FA-Se@DOX presented a faster release of DOX in the acidic environment during the initial 30 h, which was up to 83.3%. However, the release rate was just 47.8% in a normal physiological environment (pH 7.4). These acid-dependent drug release features of FA-Se@DOX are very beneficial for drug delivery systems in cancer therapy.

### 2.4. In Vitro Cytotoxicity Study

MTT assay was used to investigate the cytotoxicity of different formulations of DOX against HeLa cells in vitro. Free DOX and passive targeting nanoparticle Se@DOX were set as negative control. As shown in Figure 4B, the viability of HeLa cells exposed to various formulations of DOX gradually declined with increasing DOX concentrations. For instance, free DOX, Se@DOX, and FA-Se@DOX at the DOX concentration of 8 μg/mL obviously suppressed HeLa cells’ proliferation, and cell viability rates were 58.3%, 44.5%, and 27.6%, respectively, suggesting that FA-Se@DOX exhibited greater cytotoxicity in HeLa cells compared with free DOX and Se@DOX. The cell viability of HeLa cells was significantly lower than 50% after treatment with FA-Se@DOX at the equivalent DOX dose of 4 μg/mL; thus, such a dose was applied for further biological research. The proliferation inhibition of HeLa cells treated with drug carrier FA-SeNPs at the used dose was not obvious (Figure S1), indicating the low cytotoxicity of FA-SeNPs. The MTT results indicated that the delivery of DOX using active tumor-targeted carrier FA-SeNPs could effectively enhance the anticancer activity of DOX.

### 2.5. FA-Se@DOX Suppress the Migration and Invasion of HeLa Cells

**Figure 5:**
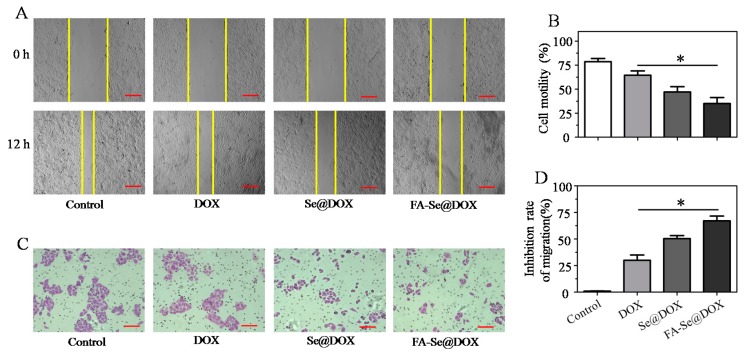
(**A**) The scratching width was observed at 0 h or 12 h after the treatment with DOX, Se@DOX, and FA-Se@DOX. Scale bar is 400 µm. (**B**) Cell motility of control, DOX, Se@DOX, and FA-Se@DOX were quantitatively analyzed. * *p <* 0.05 vs. free DOX group. (**C**) The effect of DOX, Se@DOX, and FA-Se@DOX on the migration of HeLa cells after treatment with DOX, Se@DOX, and FA-Se@DOX for 24 h. Scale bar is 200 µm. (**D**) The inhibition rate of cell migration of DOX, Se@DOX, and FA-Se@DOX were quantitatively analyzed. * *p <* 0.05 vs. DOX group.

Cell wound-healing assay was utilized to assess whether FA-Se@DOX could effectively inhibit the migration of cancer cells. As shown in Figure 5A, the wound-healing assay results showed that FA-Se@DOX decreased the migration of HeLa cells over a 12-h interval. Meanwhile, the invasion of HeLa cells was also strongly inhibited by FA-Se@DOX (Figure 5C). Furthermore, FA-Se@DOX exhibited slightly higher activity to inhibit HeLa cell migration and invasion in comparison with free DOX or Se@DOX (Figure 5B,D), indicating that FA-Se@DOX is superior to DOX and the passive targeting delivery system Se@DOX to inhibit the motility and migration of HeLa cells.

### 2.6. FA-Se@DOX Induces the Apoptosis of HeLa Cells

**Figure 6:**
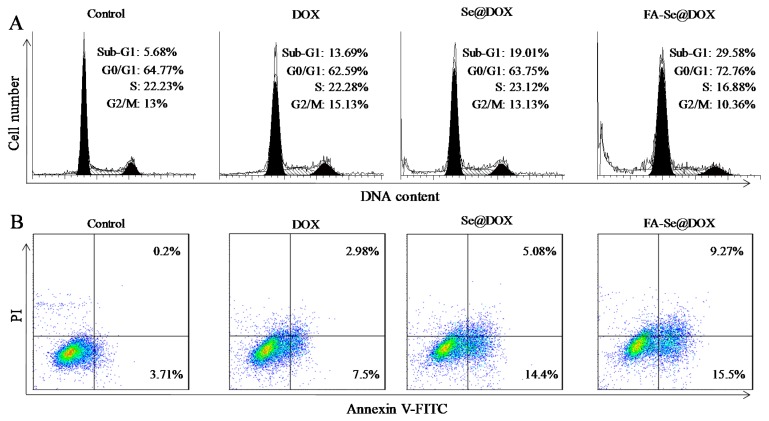
(**A**) Representative flow cytometry histograms of the cell cycle analysis of HeLa cells after incubation with various formulations of DOX for 24 h. (**B**) The apoptosis percentages analysis of HeLa cells after incubation with various formulations of DOX for 24 h.

DOX is a very effective antitumor drug and can induce cancer cell apoptosis [31]. Thus, flow cytometry was used to test whether FA-Se@DOX could exhibit greater activity to induce HeLa cells apoptosis compared with free DOX or Se@DOX. In this study, apoptosis cells with DNA fragmentations were reflected as Sub-G1 peaks. Figure 6A showed that the Sub-G1 apoptosis peak of cells in the FA-Se@DOX-treatment group was stronger (29.58%) than that of the DOX-treatment group (13.69%) and the Se@DOX-treatment group (19.01%), indicating that FA-Se@DOX exhibited a stronger capacity to induce HeLa cells’ apoptosis in comparison with free DOX or Se@DOX. However, there was no obvious difference in the cell cycle distribution among the different treatment groups. To further detect the apoptosis of HeLa cells treated with various formulations of DOX, the cells were analyzed via Annexin V-FITC/PI dual staining. As shown in Figure 6B, FA-Se@DOX-treatment obviously induced HeLa cells’ apoptosis and resulted in higher cell apoptosis rates (24.77%) compared with the cells treated with DOX (10.48%) and Se@DOX (19.48%). These results indicated that FA-Se@DOX could enhance the anticancer activity of DOX to induce HeLa cells’ apoptosis by loading DOX onto the surface of active tumor-targeting carrier FA-SeNPs.

### 2.7. In Vivo Biodistribution of Nanoparticles

**Figure 7:**
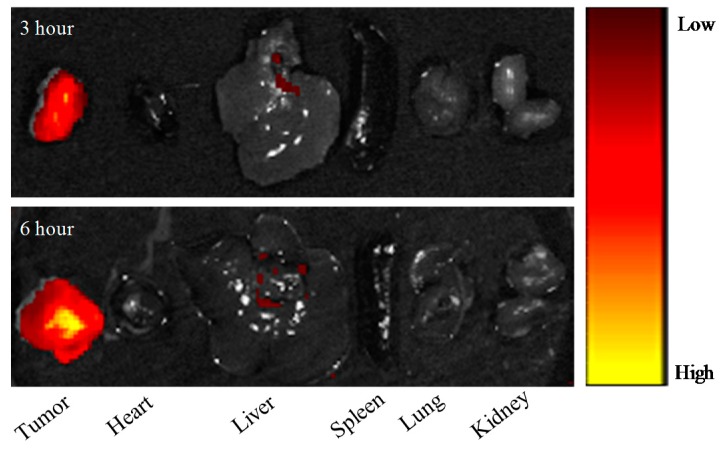
The fluorescence images of excised tumors and organs at 3 h and 6 h post-injection of FA-Se@DOX, respectively.

As effective active tumor-targeting delivery carriers, carriers should be selectively capable of delivering antitumor drugs to tumor sites for acquiring enhanced antitumor activity. Thus, the biodistribution of cy5.5-loaded FA-Se@DOX in the tumors and main organs of mice were observed by the ex vivo fluorescence imaging after 3 h or 6 h of intravenous injection. As shown in Figure 7, cy5.5-loaded FA-Se@DOX mainly accumulated in tumors in comparison with other organs. Compared with 3 h of intravenous injection, fluorescence signals became stronger after 6 h intravenous injection of cy5.5-loaded FA-Se@DOX. This result indicated that cy5.5-loaded FA-Se@DOX could achieve effective tumorous accumulation following systemic administration because of the combined contributions of EPR effects and FA-FAR specific binding.

### 2.8. In Vivo Antitumor Efficacy

**Figure 8:**
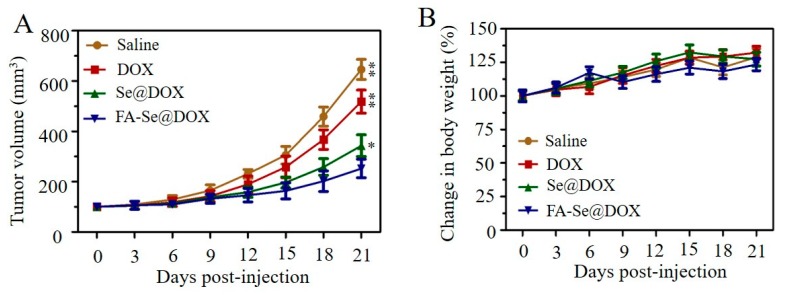
(**A**) Tumor growth curve of the xenograft nude mice bearing HeLa cells after the intravenous administration of saline and various formulations of DOX. * *p <* 0.05, ** *p <* 0.01 vs. saline group. (**B**) The body weight change of mice during the treatments.

**Figure 9:**
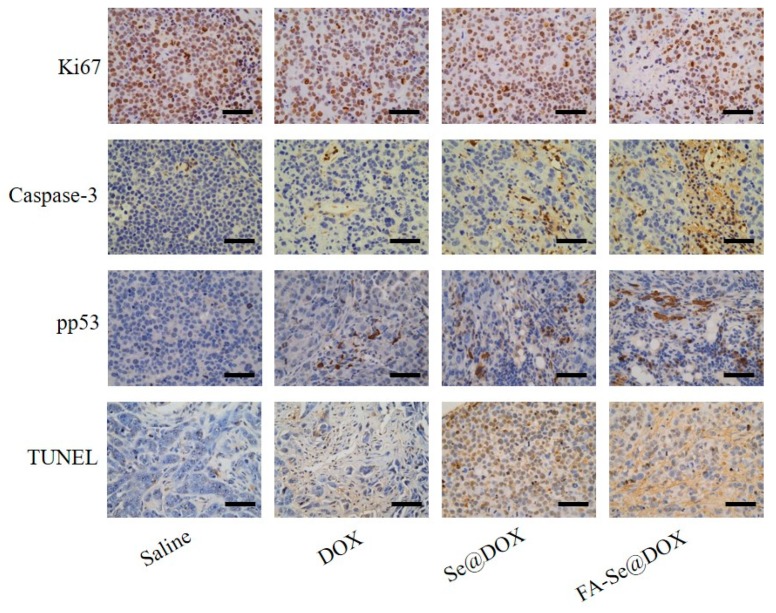
The immunohistochemical analysis of the tumor tissues from tumor-bearing mice after treatment with saline, DOX, Se@DOX, and FA-Se@DOX, respectively. Scale bar is 50 μm.

HeLa tumor xenograft was used to assess the antitumor efficacy of FA-Se@DOX. The mice were randomly assigned to four groups and then intravenously injected with FA-Se@DOX, Se@DOX, free DOX, and saline, respectively. Tumor volumes and the body weights of mice were tested every other day for up to 21 days. As shown in Figure 8A, compared to the saline-treated control group, FA-Se@DOX-treatment obviously suppressed tumor growth during the treatment time. Moreover, FA-Se@DOX was more effective in suppressing tumor growth in comparison with free DOX or Se@DOX at the same dose of DOX, proving the good antitumor efficacy of FA-Se@DOX. In addition, the body weight of mice exhibited a slight increase during the treatment period, indicating that FA-Se@DOX had no obvious side effects at the tested dose (Figure 8B). Histological studies were carried out to further elaborate the action mechanism of the enhanced anticancer ability caused by FA-Se@DOX. Cell proliferation and apoptosis in tumors were studied by Ki67, pp53, caspase-3, and terminal deoxynucleotidyl transferase dUTP nick end labeling (TUNEL) staining after treatment with FA-Se@DOX (Figure 9). Compared to the saline-treatment group, FA-Se@DOX-treatment obviously decreased Ki67-positive cancer cells, indicating that the proliferation of cancer cells was inhibited by FA-Se@DOX. As expected, the results of pp53, caspase-3, and TUNEL assays showed that FA-Se@DOX could exhibit obvious activity to induce cancer cells apoptosis in comparison with the free DOX or Se@DOX groups. The above results indicated that FA-Se@DOX presented a great potential in cervical cancer treatments via suppressing the proliferation of cervical cancer cells and inducing the apoptosis of cervical cancer cells.

**Figure 10:**
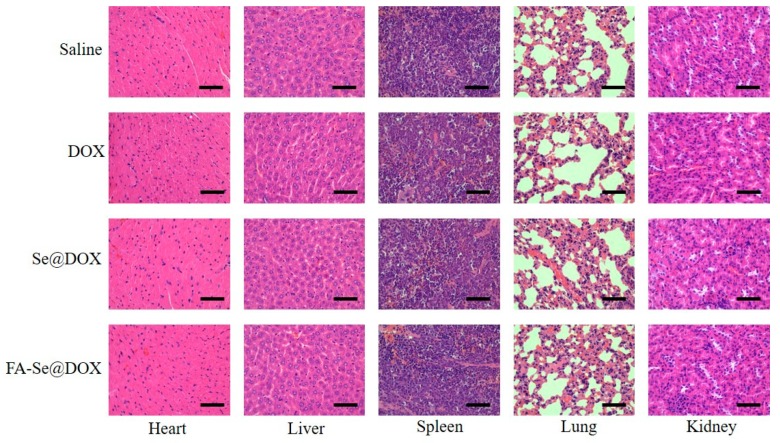
H&E analyses of heart, liver, spleen, lung, and kidney after treatment with saline, DOX, Se@DOX, and FA-Se@DOX, respectively. Scale bar is 50 μm.

To further assess the in vivo toxicity of FA-Se@DOX on main organs, an histological analysis of organs’ tissue sections was carried out via Hematoxylin and Eosin (H&E) staining. No distinct difference was found among the various treatment groups in comparison with the saline-treatment group (Figure 10), indicating that FA-Se@DOX was well tolerated at the tested dose in vivo. Analyzing these issues, the tumor-targeted delivery system FA-Se@DOX has a significant potential for effective human cervical carcinoma therapy with low systemic toxicity.

## 3. Materials and Methods

### 3.1. Materials

Folic acid, doxorubicin hydrochloride (DOX⋅HCl), sodium selenite (Na_2_SeO_3_), dimethyl sulfoxide (DMSO), ascorbic acid (Vc), Annexin V-fluorescein isothiocyanate (FITC)/PI kit, 4,6-diamino-2-phenyl indole (DAPI), and 3-(4,5-dimethyl-2-thiazolyl)-2,5-diphenyl-2-H-tetrazolium bromide, thiazolyl blue tetrazolium bromide (MTT) were purchased from Sigma-Aldrich Chemicals (Scotland, UK). The antibody was obtained from Cell Signaling Technology (Danvers, MA, USA). Penicillin-streptomycin, fetal bovine serum (FBS), and Dulbecco’s modified eagle’s medium (DMEM) medium were obtained from Gibco BRL/Life Technologies (Paisley, UK).

### 3.2. Preparation and Characterization of FA-Se@DOX Nanoparticles

SeNPs were prepared as previously reported with partial modification [32]. Briefly, 0.25 mL of Na_2_SeO_3_ (0.1 M) solution and 2 mL of vitamin C (Vc, 0.5 mM) solution were slowly added into 22.75 mL of Milli-Q water in a 50-mL beaker. Then, the solution mixtures were magnetically stirred for 30 min at room temperature to manufacture selenium nanoparticles (SeNPs). Then, folate was coated to the surface of SeNPs, and redundant components were eliminated via dialyzing for 12 h. After that, 2 mg of DOX·HCl was dissolved in 5 μL of DMSO, and then added into FA–SeNPs solutions for 8 h. At last, the high-purity FA-Se@DOX was obtained via dialyzing reaction solutions for 4 h. To investigate the in vivo biodistribution of FA-Se@DOX, a fluorescent dye cy5.5 at a final concentration of 5 μg/mL was mixed with the FA-Se@DOX solution to form cy5.5-loaded FA-Se@DOX. The chemical structures of nanoparticles were characterized by transmission electronic microscopy (TEM), dynamic light scattering (DLS) analysis, energy dispersive X-ray (EDX), and Fourier transform infrared (FTIR). The sizes of nanoparticles in the water solution were continually observed over 16 days.

### 3.3. Cell Culture

Human lung epithelial carcinoma A549 cells (folate receptor deficiency cell line) and human cervical carcinoma HeLa cells (folate receptor overexpressing cell line) were purchased from ATCC and were cultivated in DMEM with 10% FBS at 37 °C with 5% CO_2_.

### 3.4. Cellular Uptake Study

HeLa cells (5 × 10^4^ cells/well) were seeded in a 24-well plate and incubated for 12 h. Then, HeLa cells were exposed to FA-Se@DOX at a DOX dose of 4 μg/mL for 1 h, 2 h, and 4 h, respectively. Then, the cells were washed with PBS and stained with DAPI for 15 min. Subsequently, the cells were washed with PBS three times, and the cells were photographed by fluorescence microscope (Leica DMi8, Wetzlar, Germany). The cellular uptake of FA-Se@DOX in A549 cells was investigated the same way as above. The quantitative cellular uptake of DOX in various formulations was carried out by flow cytometer. Briefly, HeLa cells (5 × 10^4^ cells per well) were incubated in 12-well plates for 24 h. Then, cells were co-cultured with free DOX, Se@DOX, and FA-Se@DOX at a DOX dose of 4 μg/mL for 8 h, respectively. The cells were rinsed three times with PBS and examined by flow cytometer (FCM, BD FACSAria, San Jose, CA, USA).

The HeLa cells model was used to research the cellular uptake mechanism. Briefly, 0.5 mL of HeLa cells’ suspension at a density of 5 × 10^5^ cells/mL was incubated at 37 °C for 24 h. After that, cells were exposed to FA-Se@DOX at a DOX dose of 4 μg/mL for 4 h in the absence of an inhibitor at 4 °C, or with 50 mM of 2-deoxy-d-glucose (DOG) and 3 mg/mL of NaN_3_ or various cellular uptake inhibitors chlorpromazine (2 μg/mL), amiloride (5 μg/mL), or nystatin (4 μg/mL), at 37 °C, respectively. Then, cells were washed with PBS and gently collected. The collected cells were measured by flow cytometer (BD Bioscience, San Jose, CA, USA).

### 3.5. In Vitro Release of DOX

For in vitro release detection, 5 mg of FA-Se@DOX nanoparticles were dispersed in 5 mL of PBS solution, and then placed into a pre-swelled dialysis bag (3.5 kDa molecular weight cutoff). Then, the sealed dialysis bag was immersed into 40 mL of PBS (pH 7.4 or 5.4) with gentle agitation at 37 °C. Then, a 1-mL sample was withdrawn at different time intervals and then replaced with an equal volume of PBS. The concentration of DOX was tested by UV-vis spectroscopy.

### 3.6. MTT Assay

MTT assays were performed to test the cellular cytotoxicity of nanoparticles [33]. HeLa cells (2 × 10^4^ cells/mL) were added to a 96-well culture plate and incubated for 24 h at 37 °C. Then, cells were exposed to free DOX, Se@DOX, and FA-Se@DOX (various equivalent DOX concentrations) or with various concentrations of FA-SeNPs at 37 °C for 48 h, respectively. Then, the medium was taken away, and 100 µL of medium containing 20 µL of MTT (0.5 mg/mL) was gently added to each well, followed by incubation for another 4 h. Then, the medium was taken away, and 200 µL of dimethyl sulfoxide was added to each well. The culture plate was incubated for another 0.5 h. The absorbance of each well was tested at 570 nm by a microplate reader (Bio-Rad Laboratories, Hercules, CA, USA).

### 3.7. Wound Healing Assay

The cell migration was examined via wound-healing assay [34]. In brief, HeLa cells (5 × 10^4^ cells/well) were incubated in a 24-well plate and cultured in complete medium to reach full confluence. The monolayer cells were scratched using a sterile 10-μL pipette tip. Then, the medium was taken away and fresh DMEM containing 2% FBS were added to each well. Afterwards, HeLa cells were co-cultured with DOX, Se@DOX, and FA-Se@DOX, at a DOX equivalent concentration of 4 µg/mL, respectively, and incubated at 37 °C for 24 h. The scratch closure of the scratched monolayer cells was observed and photographed at 0 h and 12 h. The average scratched width between the sides of the wound was measured at three random areas. The migration rate was calculated according to the following equation: cell motilities (%) = [1 − (distance of scratched area at 12 h/distance of scratched area at 0 h)] × 100%.

### 3.8. Transwell Assay

Cell invasion was analyzed by Transwell chamber (8 μm). In brief, 300 μL of complete medium was added to the bottom chamber, and then, HeLa cells (1 × 10^5^ cells/mL) were seeded into the upper chambers of 24-well Transwell plates. Then, HeLa cells were incubated with DOX, Se@DOX, and FA-Se@DOX at equivalent DOX concentrations of 4 µg/mL for 24 h, respectively. After 24 h, the filter was taken away from the plate and the cells remaining on the upper filter were wiped gently. The cells that migrated to the bottom chamber were fixed with methanol and stained with crystal violet for 2 min. The average migrating cells in six independent views were photographed using a microscope.

### 3.9. Flow Cytometry Assay

The cell cycle distributions were tested by flow cytometry (BD Biosciences, San Jose, CA, USA). Briefly, HeLa cells were exposed to DOX, Se@DOX, and FA-Se@DOX at equivalent DOX concentrations of 4 µg/mL for 24 h, respectively, and washed twice with cold PBS. The pre-cooled 75% ethanol was added to the collected cells for fixation at −20 °C overnight and then stained with PI in a dark place for 30 min. The DNA contents were analyzed by Modfit software (Verity Software House, Topsham, ME, USA). Flow cytometry was also utilized to examine the effect of FA-Se@DOX on the apoptosis of HeLa cells. Briefly, HeLa cells were exposed to DOX, Se@DOX, or FA-Se@DOX at equivalent DOX concentrations of 4 µg/mL, and washed twice with PBS. Then, cells were collected and then stained with Annexin V-FITC/PI for 30 min in the dark. Finally, the stained cells were examined by flow cytometry, and the data were analyzed by FlowJo software (Treestar, Ashland, OR, USA).

### 3.10. In Vivo Biodistribution of FA-Se@DOX

The in vivo biodistribution of FA-Se@DOX was assessed in a subcutaneous human cervical carcinoma model in BALB/c nude mice (eight weeks old). Briefly, HeLa cells (1.5 × 10^7^ cells for each mice) were injected in tumor-bearing mice. After the volume of the tumors reached ~400 mm^3^, the mice were injected with cy5.5-loaded FA-Se@DOX (at an equivalent DOX of 2 mg/kg) intravenously. After 3 h or 6 h of administration, the tumors and main organs (including liver, spleen, heart, kidney, and lung) were stripped from mice, and fluorescence imaging of tumors and organs were captured by an IVIS imaging system (Xenogen, Alameda, CA, USA).

### 3.11. Xenograft Mouse Model

BALB/c nude mice (about eight weeks old) were applied to study the antitumor efficacy of FA-Se@DOX in vivo. HeLa cells (1.5 × 10^7^ cells/150 μL) were injected in the abdomens of mice subcutaneously. The mice were randomly categorized into four groups (*n* = 6) after volumes of tumors reached ~100 mm^3^. Subsequently, saline (control group), DOX, Se@DOX, and FA-Se@DOX (at an equivalent DOX of 2 mg/kg; the dose range of selenium is about 2.5 mg/kg) were intravenously injected into tumor-bearing mice once every other day, respectively. The overall treatment time was 21 days. Tumor volumes were calculated by the following formula: tumor volumes (mm^3^) = ½ × length × width^2^.

### 3.12. Hematoxylin and Eosin (H&E) Analysis

Tumors and organs were fixed with 3.7% paraformaldehyde, and then tissues were sectioned into six-µm slices. Histologic sections were prepared for H&E staining. The expression of Ki67 protein related with tumor cell growth and apoptosis-related proteins pp53 and caspase-3 was tested via immunohistochemistry. The apoptosis of tumor cells was detected by a TUNEL assay kit. The imaging of each section was photographed by a Leica DMi8 digital microscope. All of the animal experiments were carried out according to the guidelines of the Experimental Animal Center of Guangzhou Medical University and approved by Ethics Committee of Guangzhou Medical University (No 2018-274, 12 January 2018).

### 3.13. Statistical Analysis

All of the data represented mean ± standard deviations (SD). The differences between the two groups were compared using one-way analysis of variance (ANOVA). The differences were judged to be significant and highly significant at * *p* < 0.05 and ** *p* < 0.01, respectively.

## 4. Conclusions

One novel active tumor-targeting selenium nanoparticles FA-Se@DOX was successfully synthesized to deliver DOX for human cervical carcinoma treatment. FA-Se@DOX showed excellent cellular uptake in human cervical carcinoma HeLa cells, resulting in significant anticancer efficacy. FA-Se@DOX could obviously inhibit HeLa cells proliferation, and induce HeLa cells apoptosis in vitro. Furthermore, FA-Se@DOX exhibited obvious antitumor efficacy in vivo during the treatment. In addition, FA-Se@DOX showed no obvious toxicity in the main organs of tumor-bearing mice. Taken together, the tumor-targeting delivery system FA-Se@DOX provides a new strategy for human cervical carcinoma therapy.

## Supplementary Materials

Supplementary materials can be found at http://www.mdpi.com/1422-0067/19/11/3582/s1.

## Abbreviations

DLS	Dynamic light scattering
DOG	2-deoxy-d-glucose
DOX	doxorubicin
EDX	Energy dispersive X-ray
FTIR	Fourier transform infrared
FA	Folic acid
FA-Se@DOX	Selenium nanoparticles linked with folic acid and doxorubicin
Se@DOX	Selenium nanoparticles linked with doxorubicin
TEM	Transmission electronic microscopy
