# Supplementary material for: Delivery of Doxorubicin for Human Cervical Carcinoma Targeting Therapy by Folic Acid-Modified Selenium Nanoparticles
Source: Int J Mol Sci. 2018 Nov 13;19(11):3582. doi: 10.3390/ijms19113582 (PMC6274826; doi:10.3390/ijms19113582)
Supplement: Supplementary file 1 [file ijms-19-03582-s001.pdf]

Supporting Information for

“Delivery of doxorubicin for human cervical carcinoma targeting therapy by folic acid-modified selenium nanoparticles”

Yu Xia, Tiantian Xu, Mingqi Zhao, Liang Hua, Yi Chen, Changbing Wang, Ying Tang and Bing Zhu*

Virus Laboratory, Guangzhou Institute of Pediatrics, Guangzhou Women and Children’s Medical Center, Guangzhou Medical University, Guangzhou 510120, China

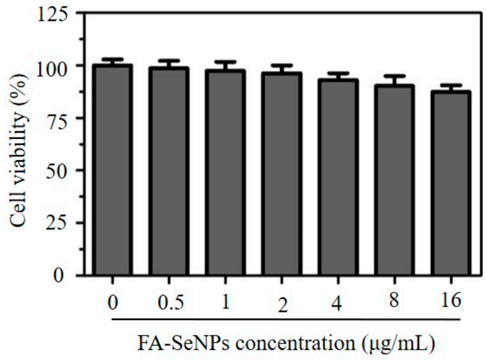

Figure S1. In vitro cytotoxicity of FA-SeNPs against HeLa cells.
